# The Role of Italy in the Use of Advanced Plant Genomic Techniques on Fruit Trees: State of the Art and Future Perspectives

**DOI:** 10.3390/ijms24020977

**Published:** 2023-01-04

**Authors:** Luca Nerva, Lorenza Dalla Costa, Angelo Ciacciulli, Silvia Sabbadini, Vera Pavese, Luca Dondini, Elisa Vendramin, Emilia Caboni, Irene Perrone, Andrea Moglia, Sara Zenoni, Vania Michelotti, Sabrina Micali, Stefano La Malfa, Alessandra Gentile, Stefano Tartarini, Bruno Mezzetti, Roberto Botta, Ignazio Verde, Riccardo Velasco, Mickael Arnaud Malnoy, Concetta Licciardello

**Affiliations:** 1 Research Center for Viticulture and Enology, Council for Agricultural Research and Economics, 31015 Conegliano, Italy; 2 Institute for Sustainable Plant Protection, National Research Council, 10135 Torino, Italy; 3 Research and Innovation Centre, Foundation Edmund Mach, 38098 San Michele all’Adige, Italy; 4 Research Center for Olive Fruit and Citrus Crops, Council for Agricultural Research and Economics, 95024 Acireale, Italy; 5 Department of Agricultural, Food, and Environmental Sciences, Marche Polytechnic University, 60131 Ancona, Italy; 6 Department of Agricultural, Forest and Food Sciences, University of Torino, 10095 Torino, Italy; 7 Department of Agricultural and Food Sciences, University of Bologna, 40127 Bologna, Italy; 8 Research Center for Olive Fruit and Citrus Crops, Council for Agricultural Research and Economics, 00134 Rome, Italy; 9 Department of Biotechnology, University of Verona, 37134 Verona, Italy; 10 Research Center for Genomics and Bioinformatics, Council for Agricultural Research and Economics, 29017 Fiorenzuola D’Arda, Italy; 11 Department of Biotechnology, University of Catania, 95124 Catania, Italy

**Keywords:** woody plants, qualitative traits, new genomic techniques, climate change resilience, genome editing, cisgenesis, intragenesis

## Abstract

Climate change is deeply impacting the food chain production, lowering quality and yield. In this context, the international scientific community has dedicated many efforts to enhancing resilience and sustainability in agriculture. Italy is among the main European producers of several fruit trees; therefore, national research centers and universities undertook several initiatives to maintain the specificity of the ‘Made in Italy’ label. Despite their importance, fruit crops are suffering from difficulties associated with the conventional breeding approaches, especially in terms of financial commitment, land resources availability, and long generation times. The ‘new genomic techniques’ (NGTs), renamed in Italy as ‘technologies for assisted evolution’ (TEAs), reduce the time required to obtain genetically improved cultivars while precisely targeting specific DNA sequences. This review aims to illustrate the role of the Italian scientific community in the use of NGTs, with a specific focus on *Citrus*, grapevine, apple, pear, chestnut, strawberry, peach, and kiwifruit. For each crop, the key genes and traits on which the scientific community is working, as well as the technological improvements and advancements on the regeneration of local varieties, are presented. Lastly, a focus is placed on the legal aspects in the European and in Italian contexts.

## 1. Application of New Genomic Techniques in Fruit Tree Species

Fruit trees represent a fundamental group of crops for the agri-food sector of Italy. In 2020, Italian fruit farming produced a turnover of more than 10 billion EUR, accounting for more than 10% of the entire agricultural sector (Faostat data, accessed on 10 October 2022). Indeed, Italy is among the top countries for the production of citrus, grapevine, pome (apple and pear) and stone (peach, cherry, plum, apricot and almond) fruits, kiwi, chestnut, and strawberry. To protect the economic value of agricultural productions from the ongoing climate change, genetic improvement of fruit trees is needed to enhance resilience and sustainability, also in light of the ongoing international sustainable development programs (i.e. New Green Deal, European Green Deal, Farm to Fork, and Paris Agreement on Climate Change) [1,2]. Furthermore, among the breeding objectives, special attention is dedicated to the expansion of the flowering and ripening calendar, pomological diversification, the extension of fruit shelf-life, and the development of resistance/tolerance to biotic and/or environmental stresses. 

It is worth noting that tree fruit species contribute to satisfying the requirements for vitamins, carotenoids and polyphenolic compounds, and fibers thanks to the consumption of fresh and derived products [3]. Fruits and vegetables are the basis for a healthy and sustainable diet, contributing to improve the quality of life for present and future generations, even though not all people are in the conditions (availability, affordability, or lack of knowledge and awareness) to consume a minimum of 900 g of fruit and vegetables each day, the doses suggested by expert bodies [4]. The fruit market costs are influenced by several factors including the incidence and severity of plant pest and pathogen attacks, as well as the susceptibility to abiotic stress such as the limited availability of water or extreme temperatures [5]. Considering the ongoing climate change, susceptibility to both biotic and abiotic stresses is predicted to increase, with important drawbacks on productivity and, therefore, an unavoidable impact on market costs [6]. Furthermore, the energy crisis is now impacting the agricultural sector, highlighting the need for more sustainable and resilient agronomic practices [7] with a reduced input demand. 

Despite their importance, fruit species are suffering from difficulties related to classical breeding techniques, in terms of financial commitment, land resources availability, and long generation times. It is important to recall that, in fruit trees, the juvenile phase can last up to 10 years, and the length is influenced by environmental and genetic factors [8]. This means that, after crossing, breeders need to wait long time to observe the ameliorated trait, especially if it concerns the fruit. For these reasons, genetic engineering may represent a valuable strategy to improve fruit crop species [9]. The most recent discoveries in the field of molecular biology have led to the development of the ‘new genomic techniques’ (NGTs) or ‘technologies for assisted evolution’ (TEAs), as they have been renamed in Italy. Compared with conventional breeding methods, NGTs allow the time required to obtain new varieties to be reduced, as well as to precisely target specific DNA sequences without altering any other trait [10]. 

Genome editing uses engineered nucleases, including meganucleases (MNs), zinc finger nucleases (ZFNs), transcription activator-like effector nucleases (TALENs) and CRISPR (clustered regularly interspaced short palindromic repeats)/Cas (CRISPR-associated)9. After the first application of ZFNs in Arabidopsis [11], this technology was also used to target endogenous genes in apples [12] and poplars [13], albeit with a limited diffusion because of its off-target effects, difficult procedures, and unsustainable costs. On the other hand, TALENs are more precise and efficient than ZFNs, but the large carriers and high cost limit their applications in plants [14]. Compared to ZFNs and TALENs, the CRISPR/Cas9 system is undoubtedly simpler, cheaper, and more efficient; this is the reason why its use was generally most diffused and otherwise adaptable also for woody plants and fruit trees. Notably, the CRISPR/Cas9 system can be designed for any genomic targets and multiplexed by adding multiple gRNAs. In fact, CRISPR/Cas9 represents an innovative technique to modify specific genome sites, as well as to introduce new genetic variants by DNA double-strand breaks (DSBs) and nonhomologous end joining (NHEJ), or homology-directed recombination (HDR) [15]. Due to the efficiency and simplicity of this system, it is increasingly being used for modifying the traits of many plants, including important crops, and for developing improved germplasm resources. The CRISPR/Cas9 machinery can be delivered to the plant cell in two different ways: as stable integration of the DNA cassette, under selective pressure for DNA integration, as transient transformation by direct mRNA delivery avoiding selective pressure [16], as guide RNA plus Cas proteins as ribonucleoprotein (RNP) complexes [17], or through virus-based vector [18]. Several issues are associated with stable transformation using the CRISPR/Cas cassette such as the random integration into the host genome, with the risk of impacting coding or important regulative regions, and the necessity to perform backcrosses in order to segregate the exogenous DNA cassette while preserving the editing event(s) [10]. For this reason, the application of DNA-free approaches exploiting protoplasts is now increasingly reported in the literature [19]. Overall, the use of NGTs represents a valid instrument to speed up the long times required by classical breeding, intervening in a surgically precise manner in target sequences, while maintaining the rest of the genetic background unaltered. In fact, this approach would allow the value of the main fruit species cultivated in Italy to be increased, improving several aspects that, to date, penalize the final products disregarding the requests of national and international sustainability programs, as well as those from consumers and producers. 

At the international level, the application of these technologies is impressive, the main players being the United States and China (https://publications.jrc.ec.europa.eu/repository/handle/JRC123830, Accessed on 9 November 2022). Looking at the most recent worldwide studies in the fruit crops sector (Table 1), in general, most of the studies focused on defense against biotic stresses. In some cases, the effort was mainly focused on a specific pathogen, such as the bacterium *Xanthomonas axonopodis* that causes the *Citrus* bacterial canker (CBC) disease in the genus *Citrus*, or the *Plum pox virus* affecting *Prunus* species, while, in other cases, such as in grapevine, a wider range of pathogens was considered. In addition, in some species, current research is directed toward the improvement of traits linked to yields, such as in kiwifruit and strawberries. 

In addition to genome editing, cisgenesis and intragenesis techniques also belonging to the NGTs allow a speed up in comparison to conventional breeding approaches. The results of cisgenic approaches are plants modified with the addition of gene(s) isolated exclusively from sexually compatible species, including introns and regulative regions (i.e. promoters and terminators) in their native sense orientation [39,40]. In the case of intragenic plants, the inserted sequence is the result of a new arrangement of genetic elements isolated from the same species or different sexually compatible ones (e.g., a gene promoter from one species and a coding sequence from another one, both sexually compatible) [41]. In both cases (cisgenic and intragenic plants), the presence of selectable markers genes is forbidden. The advantage of these approaches is that the plants obtained resemble those derived from conventional breeding approaches, without the inconvenient integration of undesired traits [42]. The latter phenomenon, known as linkage drag, is usually solved by the double pseudo-testcross strategy which can require decades in the case of woody plants with long juvenile phases. Cisgenesis and intragenesis allow the linkage drag issues to be bypassed by transferring only the desired gene(s) in a single step, preserving all the quality traits selected in elite cultivars appreciated by the final consumers.

**Figure 1:**
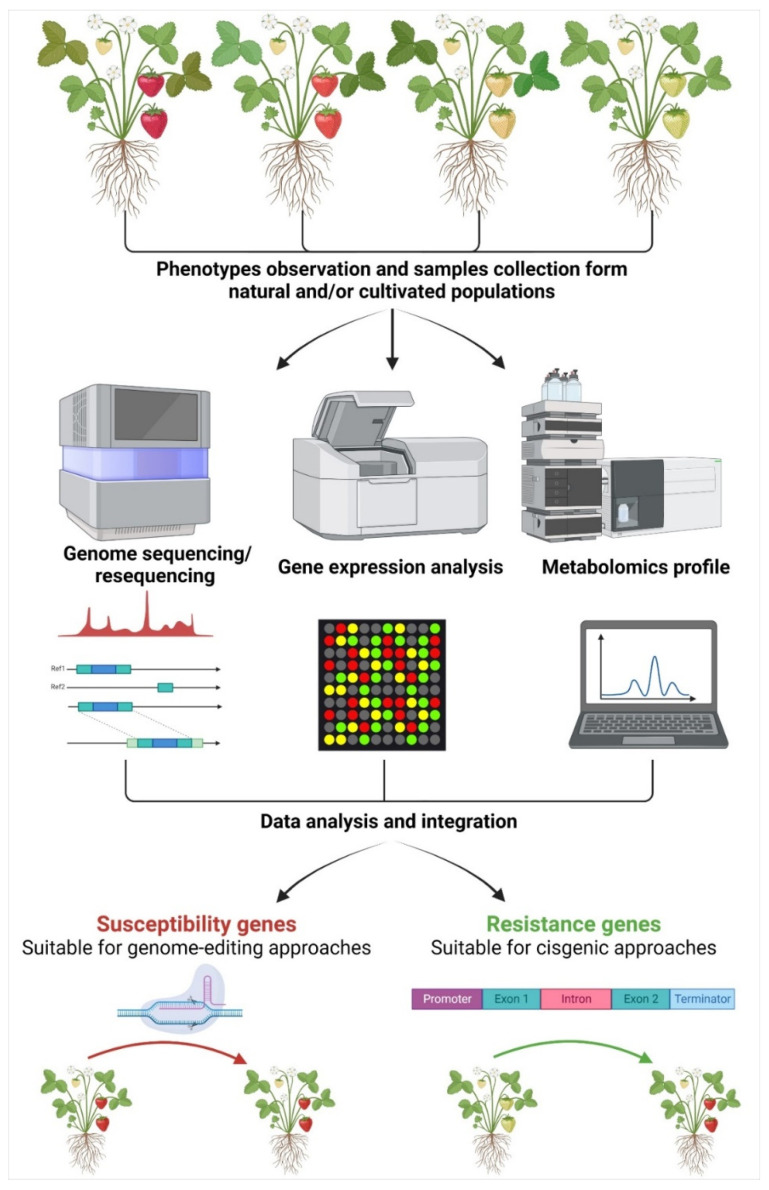
Overview of the multidisciplinary approaches to identify candidate gene(s) exploitable for genome editing or cisgenesis approaches. Starting from the natural occurring phenotypes, including both cultivated and wild genotypes, it is possible to identify genetic sequences involved in the determination of specific traits. More in detail, combining genomic analysis with gene expression and metabolomic data, it is possible to identify pathways and key genes controlling the trait(s) of interest. Once identified, if concerning negative regulator(s), such as stress-related susceptibility genes, it is possible to apply genome editing to obtain an improved genotype where the expression of such gene(s) will be knocked-out. On the contrary, the identification of positive regulator(s) can be exploited in cisgenesis approaches to obtain new genotypes expressing the resistance/tolerance-related gene(s).

Notwithstanding the potentiality of NGTs, they suffer from drawbacks that strongly limit their use. First of all, the knowledge of genes responsible for specific traits of interest is not always available. While the availability of the genome represents a starting point, it must be integrated with more accurate information such as gene annotation and knowledge of the protein interactions and cellular functions [43]. In the framework of fruit trees species, several genomes of important species have already been made available, including grapevine [44,45], citrus [46,47], peach [48,49], apricot [50,51], cherry [52,53], apple [54,55], pear [56,57], kiwifruit [58], strawberry [59,60], and chestnut [61,62]. Therefore, the knowledge on genes controlling traits of interest must be elucidated using the most modern technologies and combining data from multidisciplinary approaches to identify the best candidates (Figure 1).

**Figure 2:**
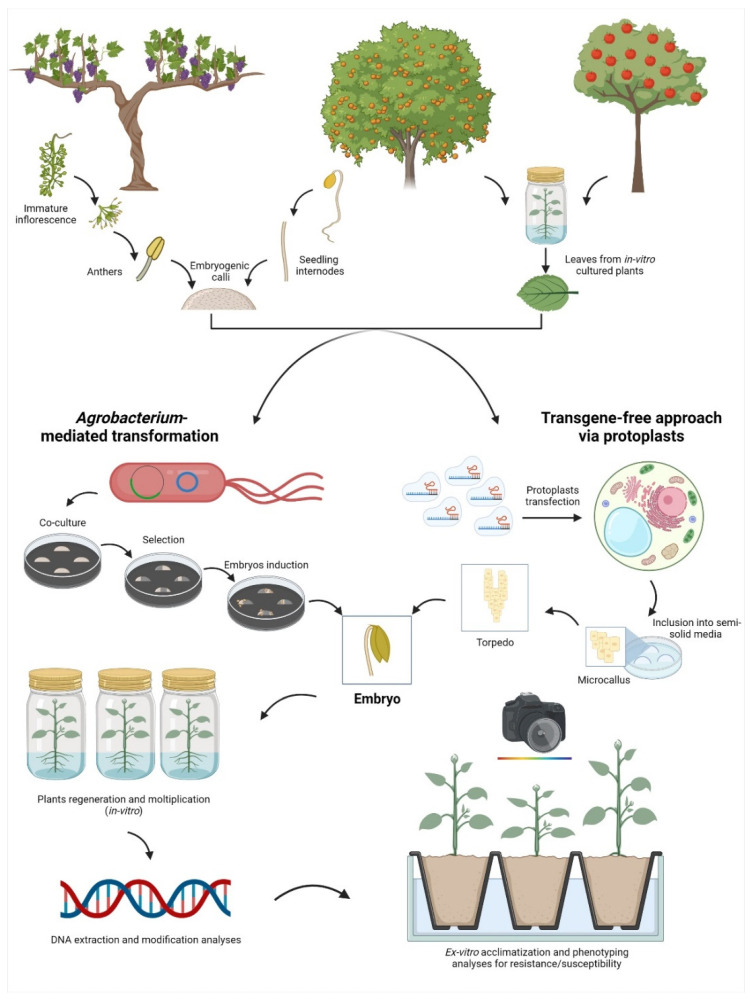
Representation of the main workflows exploited for the application of the new genomic techniques (NGTs) in some fruit crops selected as an example using somatic embryos. From left to right: in grapevine, the most exploited tissues to produce embryogenic calli belong to flowers (e.g., anthers and ovaries); in *Citrus*, hypocotyls are exploited to obtain embryogenic calli; in apple, there is no need for embryogenic calli since, if cultured in specific conditions, the leaf cells are able to regenerate the entire plant via organogenesis. Once the morphogenic competent tissue is obtained, there are two main approaches to apply NGTs: the classical method through *Agrobacterium*-mediated transformation or the isolation and transfection of protoplasts (DNA-free approach). Once the transformation/transfection event is completed, embryos are regenerated, and the genetic and phenotypic analyses are then performed.

As a second step of complexity, the ability to transform and regenerate elite fruit crop genotypes represents the most dramatic bottleneck. In fact, the selection of the best starting tissue for the transformation event, similarly to the selection of the most suitable tissue for protoplasts isolation, is not easily achievable in all fruit crops (Figure 2). 

For example, this issue is quite relevant in grapevine, where the most diffuse tissue type for transformation events and the only one available for totipotent protoplasts isolation and transfection is the embryogenic callus [63,64]. In grapevine, it can be obtained from floral tissues (e.g., anthers and ovaries) but the competency in producing embryogenic callus is genotype-specific, and many varieties are still recalcitrant [65]. Comparable, or even more challenging, is the case of peach where reports of genetically engineered plants are limited to few cases [66]. Indeed, for species such as almond and apricot, there are no studies in the scientific literature reporting the application of NGTs (Table 1). Taking these difficulties into consideration, despite the efforts made in terms of gene selection and technology implementation, many challenges are still to be addressed.

The last limiting aspect that grips the European scientific community concerns the legislative framework. Indeed, while many countries in the world issued or are adopting a regulatory framework that excludes from genetically modified organisms (GMOs) legislation those products with small mutations (small insertions or deletions, or base substitutions) induced by site-directed nucleases (e.g., CRISPR/Cas9 system), in Europe, genome-edited, cisgenic, and intragenic plants must undergo the same environmental and food and feed risk assessment as is required for the first generation of GMO plants [67]. While the internal debate is still ongoing and the European Parliament will likely discuss the possibility to deregulate at least the gene-edited plants free of exogenous DNA (see Section 6), the scientific community is working to limit as much as possible the exogenous DNA transferred into the plant genome, by also removing unwanted exogenous sequences from the final products. The latter purposes can be achieved by using constructs including site-specific recombination systems such as FLP/Frt [68,69] or Cre-loxP [70,71], which allow transgene-free genome-edited plants to be established. The elimination of the *Cas9* gene from genome-edited plants would also prevent the occurrence of mutations at untargeted loci (off-target effect) due to the unspecific recognition of off-target sites by the sgRNA–Cas9 complex [72]. 

This review aims to illustrate the role of the Italian public research in the application of NGTs thanks to the support of Italian funding, with specific focus on some of the main fruit crops, including *Citrus*, *Vitis* (as grapevine and table grape), *Pomaceae* (such as *Malus* and *Pyrus*), *Castanea*, *Fragaria*, *Prunus* (peach, apricot and cherry), and *Actinidia*. For each crop, the principal traits on which the scientific community is working and the main active projects are presented. In particular, most of the results regarding citrus, grape, strawberry, apple, pear, and kiwifruit have been produced in the framework of BIOTECH, the first project funded by the Italian Ministry of Agriculture that, from 2018 to 2023, invested around 6 million EUR to apply NGTs on several crops, including fruit trees (subprojects named *CITRUS* for *Citrus* spp., *VITECH* for *Vitis* spp. (rootstocks, wine, and table grapes), and *BioSOSFru* for stone fruit, strawberry, apple, pear, and kiwifruit. Lastly, a focus is placed on the legislative aspects within the European context.

## 2. Improving Citrus Fruit Quality by NGT Approaches—The *CITRUS* Contribution

Citrus fruits are cultivated for different purposes, mainly for fruit production (used as fresh product or processed), and for ornamental use. The production of high-quality citrus fruits (in terms of size, sugar and acidity balance, juice yield, seedlessness, and presence of bioactive compounds) represents the main objective of breeding, along with a higher tolerance or resistance toward the main biotic and abiotic stresses. The traditional breeding strategies that have been developed at CREA and at the University of Catania for lemon, sweet orange, and grapefruit are focused on clonal selection (bud or nucellar selection). The selection considers different traits, including pomological and qualitative characteristics of the fruit, plant productivity (yield and yield precocity), and resistance to biotic and abiotic stresses, and it has led to the release of different improved citrus cultivars, such as the ‘Femminello Zagara Bianca’ lemon (a vigorous and productive clone with white flowers and green young shoots), the ‘Tarocco tdv’ (a clone with a deeper pigmentation than the old Tarocco lines), and the ‘Tarocco meli’ and ‘Tarocco ippolito’ (late maturing clones with high firmness) sweet oranges [73]. New mandarin varieties and rootstocks were obtained mainly by crossing. In particular, the diploid crossing resulted in seedy new accessions, such as the ‘Sun Red’, a deeply pigmented mandarin-like fruit [74], and the ‘F6 P12′ (*C. latipes* × *Poncirus trifoliata*) rootstock [75]. Variation of ploidy was also applied allowing the obtainment of triploid seedless varieties, such as the pigmented mandarin-like ‘Mandared’ and ‘Alkantara’, the early ripening grapefruit-like ‘Bellini’ with a reduced content of furanocoumarins (responsible for the ‘grapefruit juice effect’), and the lemon-like ‘Lemox’ that bear fruits with high quality in summer [76]. These varieties represent the result of a long and hard work that Italian citrus breeders did for more than 40 years. In Citrus, mutation breeding was used more than in other tree crops, and, in the last few decades, new grapefruit, lemon, mandarin, and orange cultivars have been obtained thanks to the effort of both public and private breeding programs in different countries [77]. Huanglongbing (HLB), CBC, and Citrus black spot are the most devasting quarantine diseases that are threatening the worldwide citriculture, putting at risk the fruits production and their commercialization. These diseases are licking the European basin, giving little advantage to finding the right strategies to deal with them. Unfortunately, for *Citrus*, as well as for many other fruit tree species, conventional breeding is a long-term and expensive process, such that molecular breeding approaches and, more recently, NGTs are expected to play an important role in speeding up breeding programs. To date, very few studies have been reported about the successful application of the CRISPR/Cas9 technology; generally, they are mainly based on the use of the Golden Gate strategy. This approach was used to introduce the resistance to *Xanthomonas citri* to susceptible sweet orange and grapefruit species [78,79]. In Carrizo citrange, a genome-editing approach was developed to produce a double thorn phenotype [80]. Very recently, Alquézar and colleagues [81] induced through base editing resistance against the herbicide selection agent imazapyr, bypassing the limitations of the presence of the transgene for the selection and obtaining transgene free plants.

In the framework of BIOTECH project, *CITRUS* was addressed to take advantage of the NGTs to improve two important traits for consumer’s needs, so as to combine anthocyanins and lycopene in a unique fruit, as well as reduce seed content (in size or in number) in mandarin and mandarin-like varieties. Transformation and regeneration in *Citrus* are variety-specific; so far, most of the results have been achieved on Carrizo citrange, ‘Duncan’ grapefruit, and ‘Valencia’, ‘Pineapple’, and ‘Jincheng’ sweet oranges, whereas mandarins and clementine and, to a lesser extent, sour orange and lemon species are considered recalcitrant or less prone to be transformed (reviewed in [82]). 

### 2.1. Two Approaches to Produce Lycopene-Rich Citrus Varieties with Anthocyanins

To date, no reference has reported citrus varieties accumulating both anthocyanins and lycopene in the pulp; these traits are difficult to be combined due to the long juvenility of pummelo, grapefruit, and sweet orange, because of the apomictic nature of lycopene-rich grapefruit and sweet orange varieties, as well as that of anthocyanin-rich sweet orange. Moreover, very little information on the genetic variants responsible for the accumulation of lycopene is available, as well as the presence of locus or markers associated with the trait. The use of cisgenesis and genome editing could contribute to overcoming most of these limits. *Ruby*, a Myb-like transcription factor, was described to be responsible for the control of anthocyanin production in blood oranges, specifically for the directed association with the upstream long terminal repeat (LTR) acting as promoter, absent in common nonpigmented oranges [83,84]. The role of *Ruby* has furthermore been demonstrated in transgenic experiments, increasing the anthocyanin accumulation in nonpigmented *Citrus* species [85,86]. For our aim, we used *Ruby* as cisgene in lycopene-rich accessions (i.e., ‘Vaniglia Sanguigno’, an acidless red-orange grapefruit, and ‘Star Ruby’, a pink grapefruit). We developed two constructs [87], one consisting of a cisgenic vector for the *Ruby* gene, and a second harboring the Cas9 and sgRNA designed to induce the knockout in two *Terminal Flowering Locus* 1 (*TFL1)* genes, Cs8g15080 and Cs6g15000, with the aim to induce the precocious flowering in Carrizo and in ‘Tarocco tringale’ (an anthocyanin-rich sweet orange variety), as reported for CENTRORDIALIS in *Actinidia* [88]. Both vectors contained a FLP/FRT system to excise the cassette after a heat-shock treatment and obtain marker-free cisgenic plants, similarly to what has been described in apple [72]. The regenerated plantlets were mini-grafted onto Carrizo rootstock. For the *Ruby* construct, the PCR screening identified four ‘Star Ruby’ positive lines obtained from epicotyl transformation, and 32 ‘Vaniglia Sanguigno’ positive lines (six obtained by the transformation of cotyledons and 26 by the transformation of epicotyls); the resulting efficiency was 1.7% for ‘Star Ruby’, while that for ‘Vaniglia Sanguigno’ was 6.45% and 6% from epicotyls and seed explants, respectively [87]. For the TFL1 construct, the mutations induced by Cas9 were investigated by amplicon sequencing through high-throughput sequencing (HTS); as results, we obtained three Carrizo and three ‘Tarocco tringale’ edited for both Cs8g15080 and Cs6g15000, and six ‘Tarocco tringale’ edited only for Cs8g15080. The efficiency was 1.5% for Carrizo and a total of 6.77% for ‘Tarocco tringale’, showing for the first time the possibility of using one single guide to edit two targets in anthocyainin-rich sweet orange varieties. The promising results obtained by both constructs let us to proceed with the fusion of the two constructs. The fused plasmid would be able to introduce *Ruby* and validate the phenotype by early flowering [87].

Few studies used the genome-editing approach to improve fruit quality; for example, in tomato, the biosynthesis of lycopene was promoted by inhibiting the conversion from lycopene to β- and α-carotene [89,90]. The genome editing of β-cyclase was also used to develop the β-carotene-enriched banana variety [91]. In our case, in *Citrus*, the knockout of *β-LCY2*, through a dual-single guide approach, was aimed at producing loss-of-function mutants to induce lycopene accumulation in anthocyanin-rich sweet oranges [92]. For the first time, genome editing has been adopted to improve citrus fruit qualitative traits. Several edited anthocyanin-pigmented sweet oranges have been produced, including nonchimeric genotypes, as supported by HTS analysis. Among sweet oranges, a few studies described that anthocyanin-pigmented varieties, such as ‘Tarocco’ and ‘Maltese half-blood’, starting either from mature or from young tissues, can be regenerated and transformed [93,94]. A strong effort has also been made to optimize the protocols for those varieties belonging to sweet orange and grapefruit for which regeneration was needed. In the meantime, transformation using seed-derived tissues in sweet orange has been reported, mainly on a series of anthocyanin-rich sweet oranges never tested before [87]. Moreover, the use of mini-grafting in a nonsterile environment represents a novelty, allowing a faster recovery of transformed shoots. 

### 2.2. The Production of Seedlessness Mandarin-like Fruits

Seedlessness in *Citrus* is a complex trait under the control of several biological processes. It can be observed in parthenocarpic and stenospermocarpic varieties, such as seedless mandarin Mukaku Kishu-type [95], as well as in self-incompatible (SI) ones, such as clementine, grown in isolated blocks, far from cross-pollinators [96]. In other cases, such as ‘Satsuma’ mandarin and ‘Washington Navel’ cultivars, male or female sterility contributes to producing fruits with no seeds or a significant reduction in their number [97]. Lastly, triploids produce seedless fruits, due to their sterility [98]. Therefore, the identification of genes that can be managed to produce seedless varieties is very complicated. In *Arabidopsis* it is reported that *iku* (*IKU1* and *IKU2*) and *miniseed3* (*MINI3*) mutations specifically affected the reduction in seed size through regulating endosperm proliferation and cellularization [99,100,101]; the loss-of-function mutations in *IKU* pathway genes cause a decrease in seed size. Therefore, we used a dual single-guide approach on the homologous of *IKU1* in *Citrus* [102], hypothesizing that it could regulate seed size affecting the development of the zygotic tissues also in this species, as previously described in *Arabidopsis* [103]. So far, Carrizo citrange plantlets have been recovered after editing, and then propagated; different types of mutations (insertions, deletions, and inversions) have been obtained for both sgRNAs, and the deduced sequence of the edited *IKU* gene showed in most cases the introduction of a stop codon responsible for premature termination of the protein. Further analyses on edited plantlets are ongoing [102].

In 2020, investigating self-incompatibility (SI) systems in many different *Citrus* species, Liang et al. [104], discovered that a predominant single-nucleotide mutation called S_m_-RNase, present in self-compatible (SC) accessions, was responsible for the loss of SI in the SC accessions. In order to recover the SI mechanism in sweet orange, we developed a genome-editing vector containing a sgRNA that targets the S_m_-RNase polymorphism [102]. Transformation experiments using ‘Doppio Sanguigno’ sweet orange seedlings are ongoing.

## 3. *VITECH*: Improving Grapevine Genetics Features to Develop a Resilient and Sustainable Vineyard

Among woody crops, grapevine is one of the most important species in the EU, accounting for about 50% of the grape cultivated area in the world, with more than 3.4 million hectares in 2020 (Faostat, accessed on 5 October 2022). Furthermore, the value of this crop amounts to about 30 billion EUR in Europe, of which more than 3 billion belongs to Italy (Faostat, accessed on 5 October 2022). Conventional breeding approaches have been employed to face urgent issues related to the modern viticulture [105]. With the aim of reducing the massive use of chemical compounds in vineyards against fungal pathogens, in 1998 scientists from the University of Udine and the Institute of Applied Genomics (IGA) of Udine started the genetic improvement program which led in 2015 and 2020 to the registration in the Italian National Catalog of new fungi-resistant wine varieties (https://vivairauscedo.com/contributi/download/stato-dell-arte-2022-it.pdf, accessed on 20 December 2022). Evaluations of their agronomic traits, enological features, and field resistance to downy and powdery mildews are reported in literature [106,107,108]. In 2020, the Foundation Edmund Mach together with Civit (Consorzio Innovazione Vite) also registered four wine varieties resistant to fungal pathogens (https://www.vinievitiresistenti.it/2020/10/09/nuovi-nomi-per-le-quattro-varieta-2020-di-fem-e-civit/, accessed on 20 December 2022). Likewise, also at CREA (Arezzo, Conegliano, Turi, and Velletri), breeding activities are underway to obtain wine varieties resistant to fungi (https://www.crea.gov.it/web/viticoltura-e-enologia/breeding-vite-da-vino-arezzo-conegliano-turi-e-velletri-, accessed on 20 December 2022). Regarding table grapes, it is only since the last few decades that Italy has resumed the activity of genetic improvement by breeding aimed at obtaining table varieties featuring resistance to disease and abiotic stresses, with naturally large berry size, preferably seedless, good firmness of the pulp, and good resistance to handling, transport, and storage (https://vivairauscedo.com/contributi/download/stato-dell-arte-2022-it.pdf, accessed on 20 December 2022). Although most cultivars in Italy are with seeds (e.g., Italia, Victoria, and Red Globe), interest in seedless varieties that better respond to market needs has recently grown. Lastly, the modern viticulture requires a renewed interface between grape and soil, able to face abiotic stresses exacerbated by the ongoing climate change. Recently, scientists from University of Milano developed a breeding program focused on rootstocks in order to select new genotypes resistant to abiotic stresses, such as drought and calcareous soils, through the development and validation of genetic and physiological markers. Four new genotypes have been obtained (‘M’ series) and registered to the Italian National Catalog of Grape Varieties. Their features are well described in the literature [109,110,111]. Moreover, the University of Bologna is carrying out a breeding program for the development of rootstocks to reduce grapevine vigor (https://agrimpresaonline.it/due-nuovi-portinnesti-per-ridurre-la-vigoria-della-vite/, accessed on 20 December 2022).

In this context, *VITECH* is dedicated to improve the resilience and sustainability of grapevine (*Vitis* spp.) production by means of NGTs, pursuing the same main objectives of conventional breeding: (i) enhancing resilience to biotic stresses exploiting both cisgenesis and genome-editing strategies, (ii) using the genome-editing approach to develop elite seedless table grape cultivars (e.g., cv. Italia), and (iii) developing improved cultivars and rootstocks genotypes to cope with climate change, specifically with drought.

### 3.1. Grapevine Tolerance to Powdery and Downy Mildews

To produce pathogen-tolerant grape plants, which require lower pesticide treatments, many efforts have been focused on the development of several genome-editing approaches applied on susceptibility genes [112] and on other important genes involved in the regulation of the responses to biotrophic pathogens [113]. The loss-of-function mutation, mediated by CRISPR/Cas9, of susceptibility genes to powdery and downy mildew was the most used strategy [114,115]. In parallel, a cisgenic approach was developed to introduce resistance to *Plasmopara viticola* (RPV3-1 locus) [116] into elite wine grape cultivars (e.g., Glera, Sangiovese, Chardonnay, and Pinot noir). Due to the limitations of working with woody plants with highly heterozygous genomes and, therefore, the impossibility to undergo backcross strategies [10], a DNA-free approach through transfection of embryogenic callus-derived protoplasts by means of a CRISPR/Cas9 RNP system is highly desired. Very recently, the possibility to obtain transgene-free edited grapevine plants through the regeneration of edited protoplasts has been demonstrated in ‘Thompos seedless’, encouraging the use of genome editing for the genetic improvement of grapevine [117]. Grapevine-specific miRNAs 3632 and 3623 (not yet characterized) and miR482 (already known as involved in disease response in tomato and cotton [118,119]) regulate *NBS–LRR* disease-resistance genes. SgRNAs targeting these miRNAs have been used, in order to improve grapevine responses to pathogens. Regenerated embryos of ‘Chardonnay’ and ‘Brachetto’ cultivars are now in culture and will be analyzed for editing events. 

### 3.2. Seedlessness Table Grape

To reach the second objective, many efforts have been focused on the development of embryogenic calli from important table grape varieties (e.g., cultivars Italia, Victoria, and Red Globe) allowing the setup of an improved protocol [120]. This objective is linked to the fact that seedlessness is one of the most desired traits by consumers of table grapes. Several studies already defined the central role of an MADS-type transcription factor, *VviAGL11*, in the process of stenospermocarpy [121], which consists of an embryo abortion with consequent seed development regression after full fertilization. However, no working mechanisms have been reported yet. Additionally, the existence of specific promoter-coding sequence (CDS) combinations, that directly affect the *VviAGL11* expression level, has already been demonstrated. Transcriptomic analyses on ovule and developing seeds in seedy and seedless varieties have highlighted the role of *VviAGL11* in hormone signaling and phenylpropanoid metabolism, identifying a methyl jasmonate esterase, an indole-3-acetate beta-glucosyltransferase, and an isoflavone reductase, as direct targets of *VviAGL11*. The dominant negative effect of the mutated *VviAGL11* CDS [122] on target gene activation was molecularly validated, and a new regulatory mechanism correlating *VviAGL11* haplotype assortment and seedlessness class in grapevine was proposed [123]. The next step will be the functional characterization of *VviAGL11* through the generation of edited microvine plants, a model system which makes it possible to obtain fruits in a few months accelerating the long generation cycles of common cultivars [124].

### 3.3. Resistance to Drought

One of the projects dedicated to improve resilience to the ongoing climate change (i.e., drought), a rapid and innovative method consists of the application of spray-induced gene silencing (SIGS), to functionally validate genes in rootstocks [10]. Thanks to this approach, a gene belonging to the glutathione S-transferase family, namely, *VvGST40*, was characterized as a potential candidate for the application of gene editing to counteract drought effects. Once downregulated using the SIGS approach, the resulting plants displayed an increased abscisic acid (ABA) accumulation and an improved antioxidant arsenal limiting the negative effects of drought stress [125]. In parallel, the study of the genetic basis of stomata formation in the leaf, which is a key trait for plant response to drought and heat stress, has been initiated. The two grapevine isoforms of epidermal patterning factor-like 9, known to be a key regulator of stomata formation in model plants and cereals, were chosen as target for knockout (KO) and overexpression experiments. *Epfl9-1* KO ‘Sugraone’ mutants showed reduced stomatal density [126], and further research is ongoing to depict the role and peculiarities of *VvEPFL9-2.*

## 4. Chestnut: Genome Editing to Introduce the Tolerance to Biotic Stress

The European chestnut (*Castanea sativa* Mill.) is a woody species of high economic interest, widely cultivated in Italy, especially in Campania, Calabria, Tuscany, Latium, and Piedmont Regions. It is considered a multipurpose species for the nutritional value of its nuts, the timber quality, the role in modeling the landscape of mountain areas, and the ecological importance of its forests and orchards. At the moment, in most areas of Italy, the chestnut industry is still built on ancient orchards, often over 100 years old, since the renewal of plantings is very poor, in spite of the high nut demand from the confectionery industry and the fresh market. This is due to several causes, one being the presence of pathogens that are particularly aggressive against young plants and require intensive care in the first years after planting. Chestnut breeding has been focused on obtaining cultivars with higher yield, nut and timber quality (large nut size, easy peeling and good wood quality, fast growth), and tolerance to the major pests and pathogens of the species. Among chestnut species, *C. mollissima* and *C. crenata* are tolerant to *Phytophthora* spp. [127] and have been used to obtain hybrids bearing this trait [128]. Breeding programs carried out at INRAE, crossing *C. sativa* and *C. crenata*, produced a set of hybrid selections with a higher tolerance to Phytophthora that are currently used as rootstocks and as direct producers, despite the lower quality of nuts [129,130]. Concerning the canker blight disease, caused by *C. parasitica*, no breeding programs for obtaining improved varieties are currently underway in Europe. On the contrary, a large breeding program has been carried out in the USA to restore the species *C. dentata* by introgressing resistance genes from *C. mollissima*. Breeding programs aimed at selecting resistant genotypes to the Asian gall wasp *Dryocosmus kuriphilus* and introducing resistance in cultivars were active in Japan and Italy [131], before biological control was established. *C. sativa* is highly susceptible to two severe diseases that undermine its survival: ink disease induced by the oomycete *Phytophthora* spp., and chestnut blight caused by the fungus *Cryphonectria parasitica* [132]. Moreover, over the past 20 years, chestnut has been affected by *Dryocosmus kuriphilus* Yasumatsu, the Asian gall wasp, an insect that produces galls on the shoots of the host plant, and by the fungus *Gnomoniopsis castaneae* G. Tamietti, the nut rot agent representing a new plant health emergency [133,134]. For these reasons, currently, the chestnut breeding programs are mainly focused on increasing tolerance to pathogens using genotypes with high yield and nut quality. In [132], two candidate genes *Powdery mildew resistance 4* (*pmr4)* and *Downy mildew resistance 6* (*dmr6*) were found involved in the chestnut susceptibility response to *P. cinnamomi* and *C. parasitica*. The ongoing research is currently focused on the knockout of *pmr4* and *dmr6* genes using the CRISPR/Cas9 technology in order to obtain tolerant plants, an important goal for the success of the future chestnut industry in a highly reduced time.

### 4.1. The Genome Editing of PDS Gene as Proof of Concept

Very recently, the use of CRISPR/Cas9 technology in the *Castanea* genus was used to target the *phytoene desaturase* (*pds*) marker gene, involved in chlorophyll biosynthesis [135]; the silencing of this gene causes the appearance of an albino phenotype [136,137] and is a visual system commonly used to test the editing technique efficiency for the first time in a new species [32,138,139]. The *pds* vector was designed using the GoldenBraid assembly system, targeting two gRNAs located in two conserved regions of the chestnut *pds* gene. Somatic embryos were chosen as starting material due to their high transformation rate compared to other explants; moreover, the possibility of obtaining regenerated chimeric plants is reduced using somatic embryos as target material [29]. Nonpigmented ‘albino’ shoots obtained from in vitro cultures were associated with the successful editing of *pds* gene with an average gene efficiency of 61% for gRNA1 and 56% for gRNA2. This work opens the way for the use of the CRISPR/Cas9 system in European chestnut for breeding improvement and the valorization of ecotypes, acting on genes responsible for tolerance to biotic/abiotic stresses and quality. The innovative achievements will also provide the acquisition of new knowledge that will make it possible to extend this technique to other woody species.

### 4.2. A Successful Use of Chestnut Protoplasts to Produce Trangene-Free Edited Plants

To avoid the stable integration of recombinant DNA, the CRISPR/Cas9 RNP approach can be an innovative way to obtain transgene-free plants leading to a better acceptance of these improved plants by consumers as compared to classic GMOs [140,141]. The protoplast represents a valuable material for the transfection process due to its high permeability to exogenous DNA [142]. The first experiment conducted in chestnut reported the setup of a protoplast isolation protocol and a transfection event using the *pds* gene [143]. A high number of protoplasts/mL was obtained (4,500,000), 91% viable. The number of isolated protoplasts was close to the number of protoplasts extracted from grape and apple tissues [144,145], and higher compared to *Quercus ilex* and *Populus alba* [146]. Protoplasts were transfected with the RNP complex targeting the *pds* gene; an editing efficiency ranging from 15 to 20% was obtained, in agreement with data observed in rice (8.4–19%) and *Arabidopsis* (16%), and higher than in grapevine and apple (0.1% and 0.5–6.9%, respectively) [140,144]. Compared to the CRISPR/Cas9 traditional approach [135], the gRNA editing efficiency estimated using the same gRNA with RNPs was lower. Different regeneration media were tested to optimize the regeneration process from protoplasts, a step that represents a major bottleneck in the application of transformation techniques in chestnut. Future research will focus on obtaining protoplasts directly from somatic tissues (leaves), to preserve the genotype of the mother plant, and on improving the regeneration technique. This CRISPR RNP-based genome editing protocol represents a powerful breeding tool for chestnut and can be transferred to other woody species that present a high genome complexity and a long juvenile phase.

## 5. *BioSOSFru*: A Large Project to Improve the Main Fruit Species Qualitative Features and Response to Biotic Stresses

Italy is among the first countries in the world for cultivated area of pome fruits (apple and pear), stone fruits (peach, cherry, plum, apricot, and almond), kiwi, and strawberry. The *BioSOSFru* aims at developing superior varieties for agronomically relevant traits and resistance to biotic stresses in stone fruits, pome fruits, strawberry, and kiwifruits through cisgenesis and genome editing (i.e., CRISPR/Cas9). 

### 5.1. Apple and Pear

Genetic improvement of pome fruit trees species can be conducted through intra- or interspecific crosses, but this approach is very lengthy and not able to guarantee the maintenance of all the traits typical of a well-known cultivar. As an example, it took over 70 years to introduce scab resistance into commercial apple cultivars starting from the wild species *Malus floribunda* 821 [147]. Pyramidations for cumulating scab and fire blight resistance [148], or scab and rosy apple aphid resistance [149] have also been described in apple. Fire blight-resistant apple and pear cultivars have also been obtained through conventional breeding [150,151]; generally, it takes several years, too much if we want to try to protect these species from pests. As an alternative to crossbreeding, the conventional fruit tree genetic improvement has made extensive use of clonal selection or artificial mutagenesis, using chemical or physical mutagenic agents (i.e., γ-rays [152]). Several pome fruit varieties were obtained by clonal selection or mutagenesis with a good commercial success. No adverse effects on humans or the environment have been shown due to the use of varieties obtained by mutagenesis; however, this is a random process and, therefore, it is not possible to direct it and predict which and how many genes will be modified.

Intense research work is being carried out at the international and national level to apply TEAs to pome crops. This is not surprising, considering the versatility of these techniques, which are potentially applicable to even minor but locally important genotypes, and the speed with which they enable the development of new market-ready varieties. 

#### 5.1.1. A Cisgenic Approach to Introduce Scab and Fire Blight Resistances 

The first cisgenic apple was developed by transferring the *HcrVf2* gene for scab resistance [153], but various studies on apple were mainly devoted to the optimization of the cisgenic approach [154]. After the first successful application, other cisgenic apple plants were also obtained for inducing fire blight resistance with a gene identified in *M.* × *robusta* 5 (*FB_MR5* gene [155]). To date, there are two cisgenic apple clones under evaluation in Switzerland and in the Netherlands, obtained by transferring resistance to fire blight (from *M. × robusta* 5) and scab (from *M. floribunda*) into the ‘Gala’ variety. The gene pool available for pome fruits improvement by cisgenesis is wide since there is a lot of experience in the use of interspecific crosses in apple and pear breeding. Furthermore, the availability of *Malus*/*Pyrus* hybrids was reported, and this is opening the way for gene transfer between the two species [156]. To this extent, the successful transfer of a scab resistance gene from apple to pear was recently demonstrated and the resulting lines were resistant to the pear scab caused by *Venturia pyrina* [157]. 

In Italy, some cisgenic apple lines have been produced by inserting the putative apple scab gene *Rvi12* and putative fire blight resistance gene from *M. fusca* [158]. Then, some European pear lines putatively resistant to fire blight have been produced [159] by inserting the already characterized *FB_MR5* apple fire blight resistance gene from *M.* × *robusta* 5. Furthermore, the insertion of another putative fire blight resistance gene from *M. fusca* (*FB_Mfu10* [160,161]) into pear is also under testing. 

#### 5.1.2. The Genome Editing to Produce Apple and Pear Resistant to Fire Blight and Self-Compatible

Most of the DNA-editing experiments in apple have been conducted to optimize the protocol by knocking down genes inducing albinism (*PDS* [162]) or by promoting early flowering (*TFL1* or terminal flowering gene [32]). Analogously, successful DNA editing in pear was also recently reported by using the same genes reported in apple [32]. A precise nucleotide substitution without double-stranded breaks (base editing) was also recently demonstrated in pear; by co-editing the acetolactate synthase (*ALS*, conferring resistance to chlorsulfuron) and *PDS*, chlorsulfuron-resistant and albin lines have been obtained [33].

In Italy, the CRISPR/Cas9 system has already been successfully applied in the apple varieties ‘Golden Delicious’ and ‘Gala’ to reduce susceptibility to fire blight via editing of the *DIPM4* gene [72]. Remarkably, the CRISPR/Cas9 transgene in the fire blight improved ‘Golden Delicious’ and ‘Gala’ plants was nearly completely removed by a programmed transgene self-elimination strategy, leaving behind a minimal trace of foreign DNA. This residual DNA does not contain any protein-coding sequence, which makes CRISPR/Cas9-based gene editing in apple a promising approach for disease control. The joint silencing of three susceptibility factors (an *HIPM* and a *DIPM* gene for fire blight, and the *mlo* gene for powdery mildew) is also under testing on an apple selection that is genetically resistant to scab and rosy apple aphid in order to obtain a multi-resistant genotype [159]. 

Regarding European pear, research is currently carried out to knock-down the *RNase* gene involved in SI, or the fire blight and powdery mildew susceptibility genes described above for apple [159].

Despite recent progress, the application of CRISPR/Cas9 in fruit tree species needs to overcome some existing problems. A crucial point is to define a GMO-free system for Cas9/gRNA delivery to the plant cells. A purified Cas9 protein was used for DNA editing in isolated apple protoplasts, without involving any foreign DNA [72,144]. Unfortunately, a large-scale application of this technique is strongly hampered by the difficulties in the regeneration of apple plants from protoplasts. Modern technologies for drug delivery already available in animal model systems [163] should be considered in the future, knowing that the plant cell walls could represent an additional problem to be solved.

### 5.2. Strawberry

Strawberry is one of the most popular and appreciated fruits by consumers for its aroma and nutraceutical properties. During the last 10 years, strawberry world production increased by 40% of global production according to the Food and Agriculture Organization of the United Nations. 

Currently, strawberry genetic improvement programs are focused on the identification of varieties sustainable for the farmer and appreciated by the consumer, with increased sensorial and qualitative aspects of the fruit. The huge amount of data acquired during the last years through strawberry genotyping and mapping has increased the use of DNA information among breeding programs to identify lineage of parentals used in programmed crosses or to select seedlings. The advances in the field of genome sequencing and bioinformatics, together with the optimization of molecular strategies, such as QTL mapping and genome-wide prediction, have also led to the discovery of important agronomic loci and complex traits in the cultivated strawberry, such as those related to flowering, disease resistance, fruit quality, and yield (reviewed by Whitaker et al. [164]). However, the genetic improvement of the octoploid strawberry is often limited by the fact that some traits are controlled by multiple loci, distributed in different subgenomes, and many of the genes and molecular markers related to important agronomic characteristics are still unrevealed. These limitations could be overcome through the complementary use of NGTs, especially for the obtainment of genetically modified plants with sequences belonging to the same species or to sexually compatible ones (cisgenesis/intragenesis), as happens with traditional breeding techniques. However, one of the aspects that remains problematic in many cases in obtaining cisgenic/intragenic lines is represented by the absence of suitable cisgenic constitutive promoters, as well as of an optimized system for the selection of transformed lines by only using selectable marker or reporter genes of vegetable origin. Few examples of plant gene sequences can confer resistance to usually toxic reagents, such as herbicides represented by the 3-phosphoscichimate 1-carboxyvinyltransferase (EPSPS), which confers resistance to glyphosate, a total nonselective herbicide, when overexpressed in plants. Recently, this isolated strawberry sequence was studied in the work of Carvalho and Folta [165], as a possible nontransgenic selectable marker gene, to be used for obtaining cisgenic/intragenic strawberry plants. In this same work, several strawberry promoters were also studied to be used as constitutive promoters to replace the use of the 35S promoter of the cauliflower mosaic virus, normally used in transgenic systems. In particular, the FanAPA1-related promoters 1 and 2 and FanUBCE2 isolated from *F*. × *ananassa* were found to be candidate constitutive promoters for the expression of sequences of interest in all plant tissues. These results served as a starting point for the design and preparation of intragenic constructs aimed at constitutively expressing the recently characterized strawberry *FveFT2* gene, in both diploid and octoploid strawberries, as a non-photoperiodic florigene, capable of conferring a remontant character in seasonal flowering strawberry varieties when overexpressed [166].

#### 5.2.1. An Example of Cisgenic/Intragenic Strawberry System 

In the framework of *BioSOSFru*, one of the objectives is the introduction of the remontant trait in a seasonal flowering strawberry variety with the inclusion of only sequences of strawberry or of vegetable origin, such as *MdMYB10* of apple, which has been used as reporter gene in apple transformation trials [154]. The *FveFT2* gene was placed under the control of the FaAPA1-R2 promoter, while the *MdMYB10* and *FvEPSPS* genes were controlled by the FaUBCE2 promoter [167]. The *FveFT2* gene was also expressed without the flanking of any reporter or marker gene, because it is able to induce a re-flowering phenotype even in vitro when overexpressed in the plant, giving the possibility of identifying any transformation events without the use of marker genes [166].

#### 5.2.2. The Genome-Editing Approach Applied to Strawberry

Mutation breeding in strawberry has been carried out through conventional mutagenesis techniques in the past, by exploiting both gamma radiation and chemical mutagens [168]. Currently, point-mutagenized strawberry plants can be obtained in a more precise manner, by both stable transformation (mediated by *Agrobacterium* or direct) and transient expression to produce CRISPR/Cas9-edited plants [138,169]. However, the production of a CRISPR/Cas9-mutagenized plant excluding other genome modification by stable transformation can be time-consuming and not easily practicable in clonally propagated heterozygote crops, with the risk of losing most of the important traits of the cultivar. Protoplasts are the most widely used plant material for genome editing by instantaneous transient expression [142,144]. This method can be used to test the on-target efficiency of gRNA and the successful construction of the CRISPR vector [170]. Despite its importance as a horticultural model plant; however, there are currently very few reports of genome editing in strawberry protoplasts [171], demonstrating the potential of a highly efficient mesophyll protoplast system for transient gene expression and induction of CRISPR/Cas9-mediated genome editing. However, the regeneration efficiency of protoplast-derived calli remains strongly genotype-dependent, thus limiting the application of an easy production of protoplast-derived genome edited strawberry plants of the main commercial cultivars. 

A first application of the CRISPR/Cas9 genome-editing system in an octoploid species to characterize the function of TM6 in strawberry flower development was reported [172]. This result was achieved by using both transient expression of the sgRNA1-2/Cas9 binary vector by infiltration of a suspension of *Agrobacterium tumefaciens* into fruits, and by the stable transformation of *F.* × *ananassa* cv. Camarosa. Gaston and colleagues [166] compared the gene-editing approach with gene-silencing and gene-overexpression strategies to induce perpetual flowering in strawberry, evidencing the most efficient result in transforming a short-day cultivar into a perpetual flowering one. Recently, Gou and colleagues [171] applied CRISPR/Cas9 to knockout the *Reduced anthocyanins in petioles* (*RAP*) gene in the cultivated strawberry *F*. × *ananassa*, obtaining both T0 generation and T1 progeny (segregating the CRISPR/Cas9 transgene) with a green stem trait, demonstrating that the *RAP* gene can be a promising candidate in fruit color breeding of strawberry. However, future application of gene editing in strawberry remains limited to the availability of high-efficiency regeneration protocols from protoplasts; otherwise, the only possibility is the stable expression mediated by *Agrobacterium*-mediated transformation of somatic tissues [173].

### 5.3. Prunus

The genus *Prunus* encompasses about 250 species including important temperate fruit and nut crops such as peach, almond, apricot, plums, and cherry with a small nonduplicated genome (<300 mb) and extensive synteny and collinearity [174]. *Prunus* crops are characterized by a long juvenile phase (3–8 years) that hampers breeding procedures. Basically, *Prunus* species have been genetically improved using classical breeding methods such as parental crossing, introgression, and untargeted mutations with physical and chemical mutagens. The advent of the genomic era allowed the design of important genetic tools such as peach and cherry SNP arrays [175,176], enabling the application of marker-assisted breeding (MAB), marker-assisted introgression (MAI), and haplotype analysis [177]. The main traits addressed are related to fruit quality (such as firmness, solid soluble content, aroma, skin cracking, and split pit), flowering and maturity date, and biotic (e.g., Sharka and Powdery Mildew) and abiotic resistance in rootstocks. For auto-incompatible species such as apricot, almond, plum and cherry self-compatible cultivars were also obtained in breeding programs. The availability of genetic and genomic tools is crucial for the application of NGTs as it facilitates causal gene discovery. Since the public release of the peach genome in 2010 and its subsequent publication [48,49], several genes controlling traits of interest have been identified, mainly regarding fruit, habitus and disease resistance characteristics [177,178]. The availability of a genomic sequence also allows genome scanning for the identification of off-target regions to avoid in the choice of a sgRNA. A major challenge in *Prunus* is represented by the in vitro steps since these species are recalcitrant to regeneration and *Agrobacterium*-mediated transformation.

#### 5.3.1. Resistance to Biotic Stress

Resistance to major pests and diseases is of strategic importance in *Prunus* breeding. NGTs offer the possibility for an elite cultivar to maintain its superior genotype while introducing only the resistance by gene transfer from a donor or by de novo creating alleles, through genome editing. In the framework of *BioSOSFru*, a cisgene approach based on the *Ma* gene belonging to TNL receptors [179,180], identified in *Prunus cerasifera*, is being applied to obtain rootstocks resistant to root-knot nematodes. *Ma* confers broad-spectrum dominant resistance to the most aggressive *Meloidogyne* species, including *M. floridensis*, which has overcome the resistance induced by R genes from *P. persica* and *P. dulcis* [179,181,182]. 

Due to the lack of R genes to Sharka disease in peach [183], CRISPR/Cas9 is being applied to address recessive resistance to this detrimental virosis, targeting susceptibility genes [184,185]. Silencing of *eIFiso4E* in *P. domestica* and *eIFiso4G11* in *P. salicina*, coding for members of the eukaryotic initiation complex eIFiso4F involved in *Plum pox virus* infection, resulted in resistant plants [186]. Different regions of *eIFiso4E* are being targeted with a different single sgRNA with the intent of obtaining an allelic series of mutations [187,188]. 

#### 5.3.2. Pillar Habitus

In peach, the columnar or Pillar habitus, a homozygous recessive phenotype (*br/br*) characterized by narrow branches and a reduced canopy diameter, could potentially boost productivity by intercepting light in a more efficient way and improving dry matter partitioning [189]. The peach Pillar phenotype is due to the knockout of the *PpeTAC1* [178,190], an ortholog of the *Tiller Angle Control 1* (*TAC1*) that promotes horizontal branch growth in rice and in different plant species [191,192,193,194]. Mutations on this gene lead to compact habitus and vertical branch growth. *PpeTAC1* occurs as a single-copy gene in most plant genomes and is highly conserved. This makes it the perfect candidate to apply a genome-editing approach in peach, cherry, and apricot. Actually, a double-sgRNA CRISPR/Cas9 vector was obtained to silence the gene. As a result of the high level of conservation, the peach sgRNAs were constructed and are being used in the three species [187,188].

#### 5.3.3. Early Flowering

Stone fruit breeding is a slow process due to the unproductive juvenile phase ranging from 3 to 8 years. Shortening it and achieving fast introgression of desirable traits is a great challenge [195]. Two genes have a central position in mediating the onset of flowering: the *TFL*, a floral repressor, and the *Flowering locus T* (*FT*), a floral inducer [196,197]. *FT* overexpression and *TFL* silencing both lead to accelerated flowering [32]. The overexpression of a heterologous *FT* gene in plum led to early flowering [198]. In the peach genome, *TFL* was initially annotated as a gene with unknown function (Prupe7.G112600), and, in the framework of *BioSOSFru*, a protein sequence phylogenetic analysis clustered it with other *TFL*s, allowing its function to be inferred. To overcome the production of GMOs, a genome editing construct has been produced and used to silence *TFL* [187,188].

### 5.4. Kiwifruit

*Actinidia* spp. are deciduous and dioecious woody climbing perennial species characterized by a relatively large genome with the basic chromosome number x = 29 and ploidy variation [199]. Kiwifruit domestication started in the early 20th century, and the available cultivars of the two commercially most important species (*Actinidia deliciosa* and *A. chinensis*) are the result of more recent, with respect to other fruit species, breeding programs [200,201]. The intensive cultivation of clonally propagated kiwifruit provided the occasion for the appearance, first in 1984 and then from 2008 to date, of the pandemic pathogen *Pseudomonas syringae* pv. actinidiae (Psa), the Gram-negative bacterium responsible for bacterial canker of kiwifruit, which has in the last years caused huge economic losses for the major global kiwifruit producers, such as China, Italy, and New Zealand [202,203]. Psa is by far the most destructive disease of cultivated kiwifruit in the world, causing leaf spotting, leaf loss, bud browning and drop, fruit desiccation and shriveling, and plant death, and infected orchards can be destroyed within 2–3 years [204]. The bacterium can asymptomatically survive for long periods in the phyllosphere and host invasion occurs through the lesions or open stomata, which are considered a major entry point [205]. The host response is partly dictated by Psa, which reduces the defense capacity of the plant. Currently, disease control is based on application of a copper foliar spray for prevention, and of acibenzolar-S-methyl as inductor of natural plant resistance [206,207,208]. The application of genome editing with CRISPR/Cas9 has emerged as an effective tool for highly efficient target shooting [209]. Biotechnology offers the possibility to apply genome-editing methods and to manipulate plant response through overexpression or downregulation of endogenous genes, but efficient regeneration systems are necessary to develop these approaches; in *Actinidia* spp. they are already available for some commercial cultivars [210,211].

#### An Attempt of Application of CRISPR/Cas9 to Produce Kiwifruit Resistant to Bacterial Canker

Recently, in the framework of the BioSOSFru, some CRISPR/Cas9 vectors were developed and used for genome editing of some *A. chinensis* var. chinensis selections [212]. A gene belonging to *AP2/ERF* transcription factors was selected as a target sequence. The Cas-Designer tool was performed on the genomic sequence of the transcription factor belonging to the *AP2/ERF* family to reach all possible targets. Furthermore, for the selection of the CRISPR/Cas9 target sequence, the cDNA sequences of *AP2/ERF* genes of *A. chinensis* var. chinensis were alienated to identify the homologous region from them. These regions, as well as the conserved domain AP2, were excluded from the pool of the target sequence. In the end, four possible sgRNAs were obtained. To develop the CRISPR/Cas9 vectors with multiplex sgRNA, two intermediate expression cassettes containing the sgRNA sequence were previously constructed [213]. Leaf explants prepared as previously reported [210] were used for infection with *A. tumefaciens* carrying these vectors, and regenerated shoots were obtained. Molecular analyses were performed using specific primers designed to amplify the insert carrying the sgRNA transcriptional cassette within the CRISPR/cas9 vectors and the presence of the insertion was observed. Further molecular analyses are in progress to estimate the type and the severity of the mutations. The above-described preliminary study represents an encouraging indication on the feasibility of application of CRISPR/Cas9 vectors in *A. chinensis* in future approaches for inducing Psa resistance/tolerance. Evaluation of edited regenerated plants is in progress, either in vitro or in vivo, to characterize the response to Psa infection also through gene expression studies. This study represents a step toward the confirmation that genome editing with CRISPR/Cas9 could be a useful tool to accelerate the development of new improved varieties in fruit trees.

## 6. Regulatory Issues in Europe, with a Focus on Italy

In 2019, the European Council commissioned a study from the European Commission (EC) regarding the status of NGTs under Union law. The council aimed to investigate whether the current regulatory framework that applies to GMOs, which in 2018 was also extended to NGT products (by the European Court of Justice with the judgment in Case C-528/16), fits for such technologies that have particularly evolved over the last decade. The EC study, published in April 2021, took into consideration views on NGTs from Member States and relevant EU-level stakeholders, scientifically supported by the European Food Safety Authority and by the Commission’s Joint Research Center. The study has recorded a very high number of NGT applications underway in Europe and around the world, which could be very useful for the sustainability of the agro-food sector in the future. However, while most of the extra-European countries have adopted or are adopting legislations that differentiate many NGT products from GMOs, making their authorization for the market more streamlined and faster, the current European regulatory framework would constitute a strong obstacle to the development of NGTs in Europe [214]. On a more technical level, the EC identified substantial issues concerning traceability of some products obtained through NGTs because it is possible to analytically detect single-point mutations in a target sequence but not their origin, whether natural or induced in the laboratory [215]. Therefore, to keep pace with the scientific and technological development and favor European economic and social progress, the EC has decided to test whether the conditions exist in Europe for a change in the legislative framework on NGTs in plants. 

### 6.1. Public Consultation Promoted by the European Commission

In September 2021, the EC initiated a policy action to assess the impact of different legislative options. Alongside this impact assessment, an online public consultation was launched through the internet portal of the EC. The consultation included two phases to collect the plurality of voices of European stakeholders and citizens. In the first phase it was possible to release a free comment over 1 month (24 September 2021–22 October 2021), while the second phase (29 April 2022–22 July 2022) involved the compilation of a questionnaire concerning the issues of risk assessment, sustainability, traceability, and labeling. 

The outcome of this second phase of the consultation was published in September 2022 (https://ec.europa.eu/info/law/better-regulation/have-your-say/initiatives/13119-Legislation-for-plants-produced-by-certain-new-genomic-techniques/public-consultation_en, accessed 5 October 2022). Regarding the current rules of GMO legislation, 79% of respondents considered them inadequate for plants obtained by targeted mutagenesis or cisgenesis. Moreover, 61% of participants are in favor of a different risk assessment approach than the one applied to GMOs. In terms of sustainability, 51% were in favor of specific regulatory provisions such as regulatory incentives or actual requirements. As regards traceability, according to most of the participants, the current requirements of the GMO legislation should be revised.

The last step of this political action, expected by mid-2023, affects the definition of a legislative proposal to be submitted to the European Institutions holding legislative power, the European Parliament, and the Council. Through its representatives who sit in the European Institutions, Italy will take part in the discussion which will lead to the final adoption or rejection of the EC legislative proposal. 

### 6.2. The Position of Italy

Compared to the first phase of consultation in which Italy was the sixth European country in terms of feedback, with 2% of responses (Germany resulted the most participatory country, with 46% of responses, followed by France), in the second phase, Italy was second in terms of number of answers given (23%), behind Germany (26%) (France, with 15%, was third). 

Locally, on 12 July 2022 the Italian joint commissions on Social Affairs and Agriculture argued favorably on the possibility of conducting out field trials on plants produced with genome editing and cisgenesis techniques for experimental and scientific purposes, under the aegis of public research [216]. Indeed, Italy invested substantial effort in this sector, and in-field experimentation could strengthen its leading role in crop genetic improvement through new genomic techniques. 

## 7. Concluding Remarks and Future Perspectives

**Figure 3:**
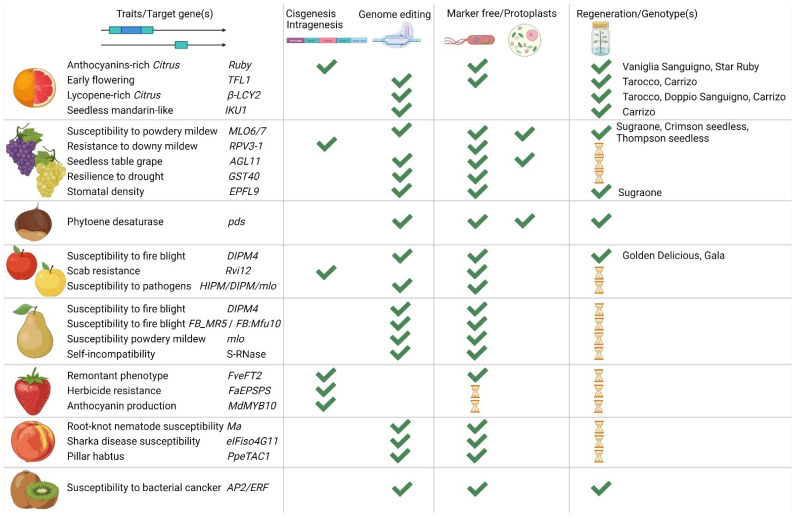
Summary of the main achievements reached by the Italian scientific community over the last years exploiting new genomic techniques (NGTs). From left to right, the species selected for genetic improvement, the gene(s) identified as target (for chestnut the selected gene was chosen as proof of concept), the technique exploited for the selected gene(s) (i.e., genome editing or cisgenesis/intragenesis), and the selected approach (i.e., *Agrobacterium*-mediated marker free transformation or DNA-free through protoplasts transfection). In the last column, we highlight if improved plants were already regenerated, and which cultivars were regenerated (published data reported in the previous sections). The hourglass represents that work is in progress.

In the last few years, Italy has invested millions of euros through national and international funding to apply NGTs on several crops, woody plants and fruit trees included. In this way, it was possible to acquire know-how on the most advanced technologies and train a new generation of scientists. Recent sources guaranteed to develop genome editing and intra/cisgenic constructs on traits the genes of which were already known, as well as on new candidate genes; moreover, new optimized protocols were successfully adapted to important varieties for the ‘Made in Italy’, mainly focused on improving regeneration capabilities from different explants (Figure 3). 

The possibility to test in field the genotypes already evaluated in the laboratory represents an opportunity to conjugate basic and applied research, encountering the needs of the national primary sector through biotechnologies. One of the most urgent scopes of application consists of protection from biotic emergencies that can compromise the survival of the local species/variety, lowering the biodiversity. In grapevine, for example, the susceptibility to powdery and downy mildew threatens the production of wine grape cultivars such as Glera, Sangiovese, Chardonnay, and Pinot noir. In this context, the use of conventional breeding cannot be sufficient to produce resistant varieties due to the admixture of the genomes. The use of genome editing allows resistance to be introduced while maintaining and protecting the quality of the elite accessions. Similarly, in *Citrus*, local lemon clones suffer, for more than a century, from Mal secco disease, thus heavily limiting cultivation. Throughout years, breeding programs were not able to introduce the resistance to the main lemon varieties, putting at risk the production of those fruits so appreciated for their nutritional and antioxidant properties. We are sure that the use NGTs can contribute to speed up the recovery of lemon growing, which has recently been suffering. Moreover, the close income of HLB in Europe would contribute to reducing, until the disappearance, the worldwide citriculture; all *Citrus* species commonly used for fruits consumption and rootstocks are susceptible. The urgency to find solutions is due to the detection of vectors transmitting the bacterium *Candidatus* liberibacter (causal agent of the disease) in Portugal and Spain (2014) and in Israel (2022). The introduction of the resistance genes in the most appreciated varieties of all the main *Citrus* species (e.g., sweet oranges, lemons, mandarins, and grapefruits) and the main rootstocks will contribute to recovering and protecting the entire citrus production. Lastly, from a qualitative point of view, the use of NGTs will contribute to protecting the mandarin ‘Tardivo of Ciaculli’, as an example, unique for its flavor and aroma, but naturally seedy and, therefore, little appreciated by consumers. If proven effective, the silencing of susceptibility genes in chestnut would greatly improve the possibility of establishing new orchards of *C. sativa* in many areas, escaping the risk of infections by canker blight and ink disease, as well as hindering the current trend toward the planting of hybrid cultivars, more tolerant to pathogens but of lower nut quality. 

Generally, this path would allow for more sustainable crops from an environmental point of view; the new varieties would allow the reduction in agrochemicals and fertilizers, lowering the needs of chemical inputs. In parallel, genetic improvement will relieve the effects of climate change, considering both biotic and abiotic stresses, with huge benefits for the agricultural sector, as well as the final consumers, while also preserving the peculiarity of the wide agrobiodiversity in Italian agriculture.

The efforts made by the Italian research community, from both the economic and the advanced know-how in research perspectives, are now freely available worldwide, allowing the exploitation of such results in many countries. This will lead to the possibility of extending the effectiveness of NGTs to agroecosystems of low- and middle-income countries, improving sustainability and resilience not only from an economic perspective but also from a social point of view. On the contrary, due to the restrictive EU and Italian regulations on the use of plants resulting from NGTs, the exploitation of the results in Italy, as well as in Europe, can suffer from huge limitations, with severe drawbacks on important currently ongoing national and international programs dealing with mitigation of climate change effects (e.g., Farm to Fork, New Green Deal, and Paris Agreement on Climate Change).

## Figures and Tables

**Table 1:** State of progress in the application of genetic engineering techniques outside Italy. Many studies report the applications of new genomics techniques and *Agrobacterium*-mediated transformation using several types of starting explants that are mainly focused on the introduction of resistance to biotic and abiotic stresses in *Citrus*, *Vitis*, *Castanea*, and *Malus*. In other species such as kiwifruit and strawberries, most recent studies are mainly directed toward the improvement of traits linked to yields and early flowering.

Genus	Species	Trait	Modified Gene(s)	Approach	Ref.
*Citrus*	*C. sinensis*	Resistance to *Citrus* canker disease	Loss of function of *CsNPR3* that represses *NPR1*	CRISPR/Cas9; Protoplast transfection with Lipofectamine	[20]
	*C. paradisi*		Mutation of an EBE in the promoter of *LOB1*	CRISPR/Cas9; *A. tumefaciens* infection of grapefruit epicotyls	[21]
	*C. sinensis*		Mutation of an EBE in the promoter of *LOB1*	CRISPR/Cas9; *A. tumefaciens* infection of epicotyls and protoplast transfection. Improved binary vector	[21]
	*C. paradisi* *C. sinensis × Poncirus trifoliata*		Loss of function of *DMR6*	CRISPR/Cas9; *A. tumefaciens* infection of epicotyls	[22]
*Vitis*	*V. vinifera*	Resistance to *Botrytis cinerea*	Loss of function of *VvWRKY52*	CRISPR/Cas9; *A. tumefaciens* infection of embryogenic callus	[23]
	*V. vinifera*	Tolerance to downy mildew caused by *Plasmopara viticola*	Loss of function of *PR4*	CRISPR/Cas9; *A. tumefaciens* infection of embryogenic callus	[24]
	*V. vinifera*	Resistance to powdery mildew caused by *Erysiphe necator*	Loss of function of *VvMLO3* and *VvMLO4*	CRISPR/Cas9; *A. tumefaciens* infection of embryogenic callus	[25]
	*V. riparia × V. rupestris*	Tolerance to Pierce’s disease and Red Blotch Disease	Disruption of the miRNA gene *TAS4a/b*	CRISPR/Cas9; *A. tumefaciens* infection of embryogenic callus	[26]
	*V. vinifera × V. berlandieri*	Control of grapevine shoot branching	Loss of function of *CCD7* and *CCD8*	CRISPR/Cas9; *A. tumefaciens* infection of embryogenic callus	[27]
	*V. amurensis*	Response to cold stress	Loss of function of *PAT1*	CRISPR/Cas9; *A. tumefaciens* infection of embryogenic callus	[28]
*Castanea*	*C. sativa*	Tolerance to Chestnut blight	Overexpression of the *CsCh3*	*Agrobacterium*-mediated transformation of somatic embryos	[29]
	*C. dentata*		Overexpression of the wheat *OxO*	*Agrobacterium*-mediated transformation of somatic embryos	[30]
*Malus*	*Malus × domestica*	Tolerance to *Botryosphaeria dothidea*	Loss of function of *CNGC*	CRISPR/Cas9; *A. tumefaciens* infection of leaf explants	[31]
	*Malus × domestica*	Early flowering	Loss of function of *TFL1*	CRISPR/Cas9; *A. tumefaciens* infection of leaf explants	[32]
	*Malus × domestica*	Proof of concept of base editing application	Base editing of *ALS* and *PDS*	CRISPR/Cas9; *A. tumefaciens* infection of leaf explants	[33]
	*M. sieversii*		Knockout of *PDS*	CRISPR/Cas9; *A. tumefaciens* infection of leaf explants	[34]
*Pyrus*	*P. communis*	Early flowering	Loss of function of *TFL1*	CRISPR/Cas9; *A. tumefaciens* infection of leaf explants	[32]
	*P. communis*	Proof of concept of base editing application	Base editing of *ALS* and *PDS*	CRISPR/Cas9; *A. tumefaciens* infection of leaf explants	[33]
*Fragaria*	*F. vesca*	Investigation of the auxin synthesis sites during fruit and root development	Loss of function of *FveYUC10*	CRISPR/Cas9; *A. tumefaciens* infection of leaf strips	[35]
*Actinidia*	*A. chinensis*	Compactness of growth habit, early flowering, and fruit development	Loss of function of *AcCEN4* and *AcCEN*	CRISPR/Cas9; *A. tumefaciens*-mediated transformation of leaf explants	[36]
	*A. chinensis*	Self-pollination and fast-flowering offspring.	Loss of function of *SyGl* and *CEN*-like genes	CRISPR/Cas9; *A. tumefaciens*-mediated transformation of leaf explants	[37]
	*A. chinensis*	Evergrowing but not early flowering phenotype	Genome editing of *AcBFT2*	CRISPR/Cas9; *A. tumefaciens*-mediated transformation of leaf explants	[38]

## Data Availability

The data presented in this review are openly available in the cited literature.
